# Early-infantile developmental and epileptic encephalopathy: the aetiologies, phenotypic differences and outcomes—a prospective observational study

**DOI:** 10.1093/braincomms/fcad243

**Published:** 2023-09-10

**Authors:** Pooja Agarwala, Bhuvandeep Narang, Thenral S Geetha, Nilesh Kurwale, Praveena L Samson, Tamanna Golani, Udita Mahadevia, Ramprasad Vedam, Sakthivel Murugan, Sagnik Chatterjee, Pradeep Goyal, Vivek Jain

**Affiliations:** Department of Pediatrics, Santokba Durlabhji Hospital, Jaipur 302015, India; Med Genome Labs, Bangalore 560099, India; Med Genome Labs, Bangalore 560099, India; Bajaj Allianz Comprehensive Epilepsy Centre, Deenanath Mangeshkar Hospital, Pune 411004, India; Med Genome Labs, Bangalore 560099, India; Med Genome Labs, Bangalore 560099, India; Med Genome Labs, Bangalore 560099, India; Med Genome Labs, Bangalore 560099, India; Med Genome Labs, Bangalore 560099, India; Statistician, Department of Economics, Banaras Hindu University, Varanasi 221005, India; Vardhman MRI Centre, Santokba Durlabhji Hospital, Jaipur 302015, India; Department of Pediatric Neurology, Neo Clinic Children’s Hospital, Jaipur 302019, India

**Keywords:** developmental and epileptic encephalopathy, phenotypes, aetiologies, genetic evaluation, outcome

## Abstract

In this study, we have evaluated the underlying aetiologies, yield of genetic testing and long-term outcomes in patients with early-infantile developmental and epileptic encephalopathies. We have prospectively studied patients with seizure onset before 3 months of age. Based on the clinical details, neuroimaging, metabolic testing and comprehensive genetic evaluation, patients were classified into different aetiological groups. The phenotypic differences between genetic/unknown groups and remaining aetiologies were compared. Factors that could affect seizure control were also assessed. A total of 80 children (M:F ratio—1.5:1) were recruited. The median seizure onset age was 28 days (range, 1–90 days). The aetiologies were confirmed in 66 patients (83%). The patients were further classified into four aetiological groups: genetic (50%), structural (19%), metabolic (14%; all were vitamin responsive) and unknown (17%). On comparing for the phenotypic differences between the groups, children in the ‘genetic/unknown’ groups were more frequently observed to have severe developmental delay (Odds Ratio = 57; *P* < 0.0001), autistic behaviours (Odds Ratio = 37; *P* < 0.0001), tone abnormalities (Odds Ratio = 9; *P* = 0.0006) and movement disorder (Odds Ratio = 19; *P* < 0.0001). Clonic seizures were more common in the vitamin responsive/structural groups (Risk Ratio = 1.36; *P* = 0.05) as compared to patients with ‘genetic/unknown’ aetiologies. On the contrary, vitamin responsive/structural aetiology patients were less likely to have tonic seizures (Risk Ratio = 0.66; *P* = 0.04). Metabolic testing was diagnostic in three out of 41 patients tested (all three had biotinidase deficiency). MRI was abnormal in 35/80 patients (malformation observed in 16/35; 19/35 had non-specific changes that did not contribute to underlying aetiology). A molecular diagnosis was achieved in 53 out of 77 patients tested (69%). Next-generation sequencing had a yield of 51%, while microarray had a yield of 14%. *STXBP1* was the most common (five patients) single-gene defect identified. There were 24 novel variants. The mean follow-up period was 30 months (range, 4–72 months). On multivariate logistic regression for the important factors that could affect seizure control (seizure onset age, time lag of first visit to paediatric neurologist and aetiologies), only vitamin responsive aetiology had a statistically significant positive effect on seizure control (*P* = 0.02). Genetic aetiologies are the most common cause of early-infantile developmental and epileptic encephalopathies. Patients in the genetic/unknown groups had a more severe phenotype. Patients with vitamin responsive epilepsies had the best probability of seizure control.

## Teaser

Agarwala *et al.* report that in babies with early (<three months age) onset epileptic encephalopathy, after comprehensive metabolic, neuroradiological and genetic evaluation, the most common cause is an underlying genetic defect. Children with vitamin responsive epilepsies had the best probability of seizure control and good long-term neurodevelopmental outcome.

## Graphical Abstract

**Graphical Abstract:**
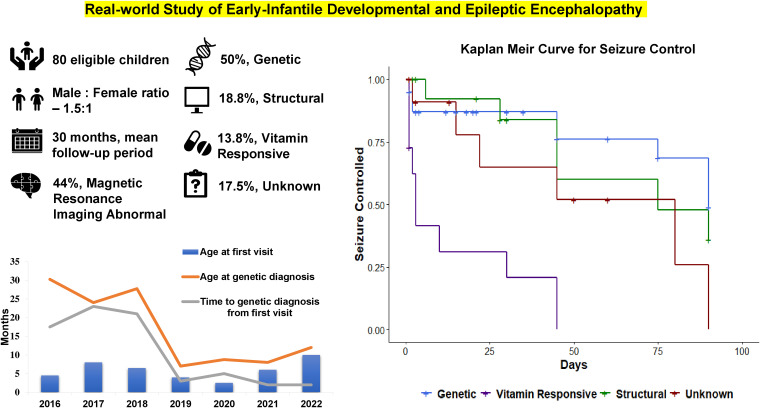

## Introduction

Developmental and epileptic encephalopathy (DEE) is a severe form of epilepsy syndrome associated with developmental impairment related to both the underlying aetiology and the epilepsy.^1^ Early-infantile developmental and epileptic encephalopathy (EIDEE) is a subgroup of DEE, where the onset of seizures is before 3 months of age.^2^ The identification of the specific underlying aetiology (channelopathy/vitamin deficiency/structural) can sometimes guide to initiate specific treatment for seizure control.^3^

The initial evaluation of children with EIDEE includes a review of medical history, physical examination, neuroimaging [magnetic resonance imaging (MRI)] and, in some cases, a therapeutic trial of specific vitamins namely pyridoxine, pyridoxal 5′-phosphate (P5′-P), folinic acid and/or biotin. Occasionally, the cause of EIDEE can be identified with this initial assessment and intervention. However, majority will also require a detailed genetic evaluation. The molecular testing strategies in these patients have varied from epilepsy gene panels to whole exome or genome sequencing.^4–9^

Even though studies on the genetic causes of EIDEE have been extensively published,^6–9^ research work on a more comprehensive evaluation to identify and phenotypically compare the heterogenous aetiologies of EIDEE is lacking.

This study was planned to particularly fill this knowledge gap. We had performed a comprehensive clinical, neuroimaging and genetic evaluation of all our eligible EIDEE patients. The patients were also followed up prospectively after enrolment. This enabled us to do a better phenotyping of the clinical profile and confirm the long-term outcome for seizure control and survival. We have also compared the clinical features that could help to distinguish between different aetiologies of EIDEE.

## Materials and methods

### Patient selection

This study was conducted at the paediatric neurology clinic at Santokba Durlabhji Hospital, a private tertiary care teaching hospital in Jaipur, North India. Due to limited availability of paediatric neurology services in public healthcare system, our centre is one of the major secondary and tertiary care referral centres for the city of Jaipur, in addition to being a tertiary centre for outlying districts (covering a population of ∼10 million people). Children were recruited prospectively between January 2016 and December 2021.

All patients included in this study met the following criteria for EIDEE:^1,2^ epilepsy onset before 3 months of age with frequent seizures; abnormal neurological examination with developmental impairment and abnormal interictal EEG (at presentation or in follow-up).

**Figure 1:**
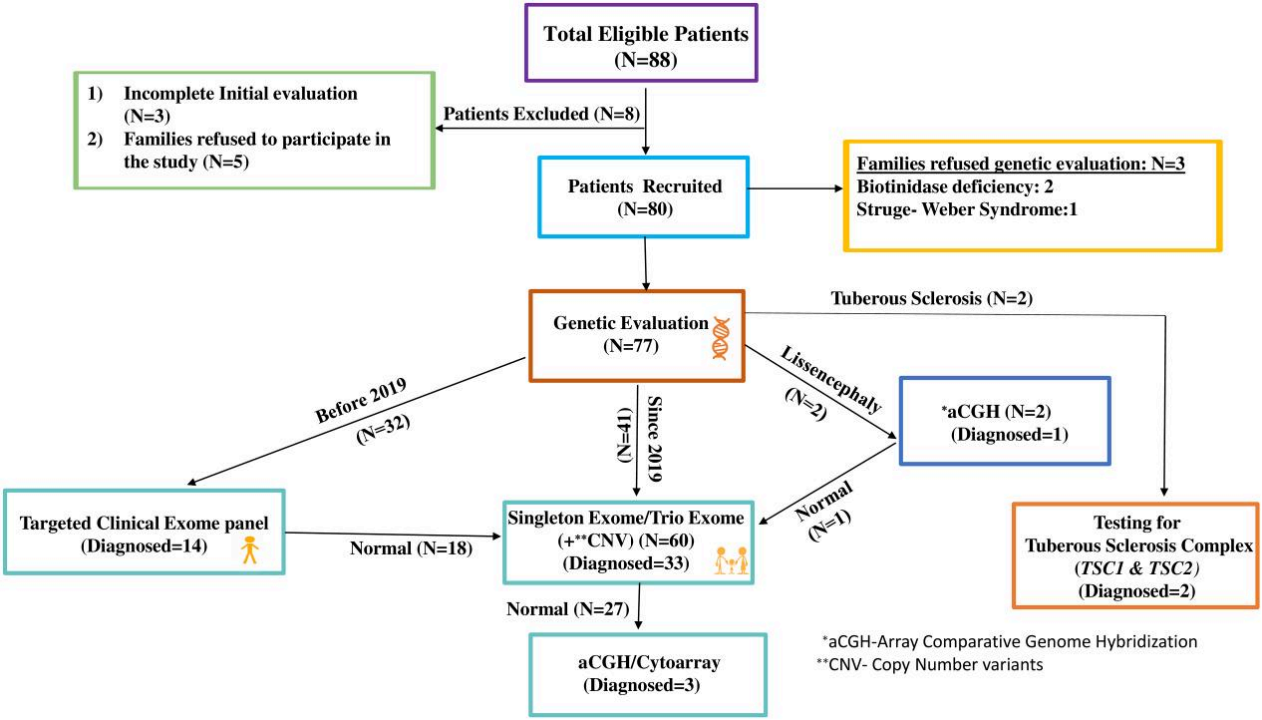
Flowchart of recruitment and steps of genetic evaluation for eligible patients.

Patients with epilepsy of infancy with migrating focal seizures (EIMFS) with seizure onset before 3 months of age were also included in this study. In addition, three families who had consented to participate in the study but refused genetic evaluation [two with enzymatically confirmed biotinidase deficiency and one with Sturge–Weber syndrome (SWS)] were also included in the final analysis (Fig. 1).

Those excluded from this study include children without family consent for participation in the study, patients with acute reactive seizures during a metabolic crisis of an inborn error of metabolism (IEM) and patients with an acquired cause (like birth asphyxia, neonatal hypoglycaemia, meningitis, perinatal stroke and haemorrhage). As this study was planned to specifically look at severe epilepsy syndromes commonly presenting before 3 months age, patients with infantile epileptic spasms syndrome as the first presentation were not included in the present study.

### Clinical details

Along with perinatal history and age at first visit to the paediatric neurologist, the following information was specifically sought at the initial consultation and reconfirmed at the follow-up visits:

- Age of seizure onset, which was further subdivided into as follows: (a) onset between 0 and 7 days; (b) between 8 and 30 days; and (c) between 31 and 90 days.
- Seizure type (mainly from history and if available home videos and prolonged video telemetry recordings). The seizure types were reclassified prior to submission, based on the recent International League Against Epilepsy (ILAE) classification for neonatal seizures and ILAE classification for epilepsy syndromes with onset in neonatal period and infancy.^2,10^
- Clinical examination specially to confirm tone abnormalities and the presence or absence of movement disorder.

### Initial investigations

Brain MRI and EEG were done on all patients. All MRI scans at our centre were performed on a 3-Tesla machine. If a child had a 1.5-Tesla brain MRI performed prior to the first visit with standard imaging protocols, neuroimaging was not repeated. All MRI scans were reviewed by the paediatric neurologist and a radiologist with interest in paediatric neuroradiology. Patients (except those with a malformation or dysplasia) also had a basic metabolic screening that included serum lactate, blood ammonia and blood tandem mass spectrometry for 49 common inborn errors of metabolism (fatty acid oxidation defects, aminoacidopathies and organic acidurias). If there was a clinical suspicion, biotinidase enzyme analysis was also done.

### Genetic evaluation

Next-generation sequencing (NGS) was done to identify the disease causing variants (Fig. 1). The detailed methodology is given in the Supplementary File.

In brief, before 2019, a targeted clinical exome panel was performed initially in all eligible patients (Fig. 1). This is a customized targeted panel consisting of ∼6000 genes including all the characterized Mendelian genes and genes from literature for inherited disease phenotypes with both autosomal dominant and recessive inheritance covering the exons and exon–intron flanks. If a variant was identified on the targeted clinical exome panel, confirmation was done with Sanger sequencing and parental testing.

In patients seen from 2019 onwards, singleton (proband only) exome sequencing (ES)/trio ES (analysed as trio along with parental samples) was performed directly (Fig. 1). Singleton/trio ES was also performed in patients with a non-diagnostic targeted clinical exome panel. Exome Research Panel from Integrated DNA Technologies and Whole exome panel from Twist Biosciences were used for performing singleton ES/trio ES.

The annotated variants were analysed using Varminer (in-house analysis tool) and interpreted based on the American College of Medical Genetics (ACMG) 2015 guidelines.^11^ ‘All pathogenic, likely pathogenic and variants of unknown significance (VUS) with strong clinical match were considered causative for an underlying genetic aetiology’.

In two patients with clinical suspicion of tuberous sclerosis, Sanger sequencing was performed directly for tuberous sclerosis complex (*TSC*) *1* and *2* genes (Fig. 1). In patients who remained without an aetiological diagnosis after NGS (Fig. 1) or had lissencephaly, the array comparative genomic hybridization (aCGH) analysis (Affymetrix CytoScan™ 750K) or Global Screening Array (cytoarray) with Cytogenetics (GSA Cyto) was performed. Detailed testing methodology is given in the Supplementary File.

### Aetiological classification

Following the clinical evaluation, neuroimaging, metabolic and genetic results, patients were categorized into aetiological groups based on the ILAE 2017 classification.^1^ These groups were as follows:

- EIDEE-genetic
- EIDEE-structural (or malformative), which was further subdivided into as follows:
  - Structural-genetic
  - Structural-unknown (patients with cortical malformation/dysplasia and a normal genetic evaluation)
- EIDEE-metabolic [including vitamin responsive (VR) patients]
- EIDEE-unknown (normal cranial MRI, metabolic and genetic evaluation with no response to trial of pyridoxine and/or P5′-P).

The phenotypic differences were compared between genetic/unknown groups and EIDEEs with ‘potentially treatable’ aetiologies (metabolic/structural).

### Follow-up data

Patients were recruited through the end of December 2021, and follow-up data were collected till July 2022. On follow-up visits, the seizure control was confirmed. If families were unable to visit the hospital, this information was collected telephonically. All patients were routinely reviewed for their neurodevelopmental progress and co-morbidities. Age-appropriate psychometric evaluation (Intelligence Quotient: Malin Intelligence Scale for Indian children; Developmental Quotient: Paediatric Developmental screening test) was performed on patients who could co-operate. Based on the scores, patients were subdivided into two groups: mild to moderate or severe developmental delay (DD)/intellectual disability (ID). Children who could not be assessed due to significant neurodevelopmental issues were considered to have severe delay based on clinical assessment by the treating paediatric neurologist. Children older than 18 months of age were also assessed for autistic behaviours, using age-appropriate standardized assessment tools (modified checklist for autism in toddlers below two years age; childhood autism rating scale for children above two). Seizure control and number of anti-seizure medications (ASMs) used were also confirmed at the last follow-up contact. Children were considered to have seizure control if they were seizure-free for at least three times the longest interseizure interval at the last follow-up contact or had not had seizure recurrence for at least three months prior to the last follow-up contact, whichever was longer.

### Statistical analysis

Data were compiled/collated in Microsoft Excel, and descriptive statistics were used to characterize patients. Further comparisons between clinical variables and outcomes of aetiological groups were conducted using the chi-square (χ^2^) test for proportional difference and two-sample proportion test. Mann–Whitney U-test was used to evaluate median difference in time lag to genetic diagnoses, for children evaluated before and after 2019. Multivariate logistic regression was also applied for assessing the factors affecting seizure control. Kaplan–Meier curves were drawn to determine the probability of seizure-free period. Graphs were drawn with GraphPad Prism 9.0.

### Ethics

The study was approved by the local institutional ethics committee (IEC/2016/12). Informed written consent was taken from families for participation in the study and for genetic evaluation.

## Results

### Patient profile

A total of 88 patients with EIDEE were seen in the study period, of which 80 were eligible to participate in the study (Fig. 1). Four of the patients from this cohort (‘Case nos. 11, 25, 26 and 49’) had already been published during the study period (Tables 1–3).^12,13^ An update on their follow-up outcomes has been presented in this study.

**Table 1:** List of pathogenic variants

S.N	Seizure onset (days)/gender	Neuroimaging	EIDEE subtype	Sequence ID	Gene	Variant description	Zygosity and segregation	Literature
1	30/F	Normal	Genetic	NM_172107.4	*KCNQ2*	c.881C>T (p.Ala294Val)	Heterozygous/*de novo*	PMID: 23692823
2	1/M	Corpus callosal dysgenesis	Genetic	NM_014023.4	*WDR37*	c.374C>T (p.Thr125Ile)	Heterozygous/*de novo*	PMID: 31327510
3	60/M	Normal	Genetic	NM_015981.4	*CAMK2A*	c.857C>A (p.Thr286Asn)	Heterozygous/likely *de novo*	PMID: 29100089
4	1/M	Normal	Genetic	NM_001032221.6	*STXBP1*	c.1074C>A (p.Tyr358Ter)	Heterozygous/*de novo*	This study
5	15/M	Normal	Genetic	NM_001032221.6	*STXBP1*	c.1439C>T (p.Pro480Leu)	Heterozygous/*de novo*	PMID: 21770924
6	30/F	Thin corpus callosum	Genetic	NM_001163435.3	*TBCK*	c.1290del (p.Arg431GlufsTer9)	Homozygous/parents heterozygous	This study
7	4/M	Thin corpus callosum	Genetic	NM_016373.4	*WWOX*	c.790C>T (p.Arg264Ter)	Homozygous/parents heterozygous	PMID: 30356099
8	21/M	Normal	Genetic	NM_016373.4	*WWOX*	c.790C>T (p.Arg264Ter)	Homozygous/parents heterozygous	PMID: 30356099
9	15/M	Normal	Genetic	NM_020822.3	*KCNT1*	c.2849G>A (p.Arg950Gln)	Heterozygous/*de novo*	PMID: 26122718
10	12/M	Cerebral atrophy	Genetic	NM_004113.6	*FGF12*	c.341G>A (p.Arg114His)	Heterozygous/*de novo*	PMID: 27164707
11^a^	45/M	Normal	Genetic	NM_001032221.6	*STXBP1*	c.1610T>C (p.Leu537Pro)	Heterozygous/*de novo*	PMID: 33196034
12	2/F	Left frontal cortical dysplasia	Genetic	NM_172107.4	*KCNQ2*	c.619C>T (p.Arg207Trp)	Heterozygous/*de novo*	PMID: 11572947
13	30/F	Cerebral atrophy	Genetic	NM_001323289.2	*CDKL5*	c.858C>A (p.Tyr286*)	Heterozygous/*de novo*	PMID: 27848944
14	4/M	Normal	Genetic	NM_001199107.2	*TBC1D24*	c.636G>A (p.Trp212Ter)/c.404C>T (p.Pro135Leu)	Compound heterozygous^b^	PMID: 28663785
15	3/F	Normal	Genetic	NM_016188.5	*ACTL6B*	c.688_689insT (p.Ala230ValfsTer20)	Homozygous/parents heterozygous	This study
16	45/F	Normal	Genetic	NM_152296.5	*ATP1A3*	c.2482G>A (p.Glu828Lys)	Heterozygous/*de novo*	This study
17	**7**/F	Cerebral atrophy	Genetic	NM_013245.3	*VPS4A*	c.298G>A (p.Glu100Lys)	Heterozygous/*de novo*	This study
18	90/M	Cortical tubers	Structural-genetic	NM_000368.5	*TSC1*	c.2886_2707del(p.leu896ile fsTer2)	Heterozygous/*de novo*	This study
19	90/M	Lissencephaly	Structural-genetic	NM_001195553.2	*DCX*	c.814C>T (p.Arg272Ter)	Heterozygous/*de novo*	PMID: 10369164
20	6/F	Multilobar dysplasia	Structural-genetic	NM_001242896.3	*DEPDC5*	c.1394del (p.Val466CysfsTer51)	Heterozygous/asymptomatic heterozygous father	This study
21	30/M	Multilobar dysplasia	Structural-genetic	NM_001242896.3	*DEPDC5*	c.3892delG, p.Glu1298ArgfsTer38)	Heterozygous/*de novo*	This study
22	1/M	Normal	VR	NM_001182.5	*ALDH7A1*	c.1556G>A (p.Arg519Lys)	Homozygous/parents heterozygous	PMID: 23925287
23	30/F	Normal	VR	NM_001370658.1	*BTD*	c.98_104delGCGGCTGinsTCC (p.Cys33PhefsTer36)	Homozygous/parents heterozygous	This study

a Case no. 11 has been previously published with our collaborators.^12^

b Parents heterozygous for each.

**Table 2:** List of likely pathogenic variants

S.N	Seizure onset (days)/gender	Neuroimaging	EIDEE subtype	Sequence ID	Gene	Variant description	Zygosity and segregation	Literature
24	21/F	Normal	Genetic	NM_001330260.2	*SCN8A*	c.4377C>G (p.Phe1459Leu)	Heterozygous/*de novo*	This study
25^a^	1/F	Cerebellar hypoplasia	Genetic	NM_001040142.2	*SCN2A*	c.638T>G (p.Val213Gly)	Heterozygous/*de novo*	PMID: 33196034
26^a^	75/M	Normal	Genetic	NM_007327.4	*GRIN1*	c.1976G>A (p.Arg659Gln)	Heterozygous/*de novo*	PMID: 33196034
27	60/M	Normal	Genetic	NM_001032221.6	*STXBP1*	c.250G>A (p.Val84Ile)	Heterozygous/*de novo*	This study
28	15/M	Normal	Genetic	NM_020822.3	*KCNT1*	c.1421G>A (p.Arg474His)	Heterozygous/*de novo*	PMID: 23086397
29	45/F	Bilateral hippocampal diffusion restriction	Genetic	NM_001165963.4	*SCN1A*	c.656G>C (p.Arg219Thr)	Heterozygous/*de novo*	PMID: 29655203
30	35/M	Dysmyelination, temporal cysts	Genetic	NM_014239.4	*EIF2B2*	c.922G>A (p.Val308Met)	Homozygous/parents heterozygous	PMID: 21307862
31	2/F	Normal	Genetic	NM_172107.4	*KCNQ2*	c.1639C>T (p.Arg547Trp)	Heterozygous/likely *de novo*	PMID: 23360469
32	90/F	Cerebral atrophy	Genetic	NM_001032221.6	*STXBP1*	c.88–2A>C (3′ splice site)	Heterozygous/*de novo*	PMID: 29655203
33	30/F	Normal	Genetic	NM_006759.4	*UGP2*	c.61A>G (p.Met21Val)	Homozygous, parents heterozygous	PMID: 31820119
34	90/M	Normal	Genetic	NM_024678.6	*NARS2*	c.1382A>T (p.Asn461Ile)	Homozygous/parents heterozygous	This study
35	75/F	Lissencephaly	Structural-genetic	NM_014975.3	*MAST1*	c.686G>C (p.Arg229Pro)	Heterozygous/*de novo*	This study
36	1/F	Normal	VR	NM_001182.5	*ALDH7A1*	c.575C>A (p.Ala192Glu)	Homozygous/parents heterozygous	This study
37	1/F	Normal	VR	NM_001182.5	*ALDH7A1*	c.1411_1412insG (p.Leu471ArgfsTer4)/c.187G>T (p.Gly63Ter)	Compound heterozygous/parents heterozygous for each	PMID: 32593896/PMID: 30043187
38	10/F	Normal	VR	NM_001182.5	*ALDH7A1*	c.575C>A (p.Ala192Glu)	Homozygous/parents heterozygous	This study
39	1/F	Normal	VR	NM_018129.4	*PNPO*	c.685C>T (p.Arg229Trp)	Homozygous/parents heterozygous	PMID: 15772097

a Case nos. 25 and 26 have been previously published with our collaborators.^12^

**Table 3:** List of variants of unknown significance (VUS)

S.N	Seizure onset (days)/gender	Neuroimaging	Gene/sequence ID	Variant description	Zygosity and segregation	Literature
40^a^	90/M	Normal	*ALG2* NM_033087.4	c.1097C>T (p.Pro366Leu)	Homozygous/parents heterozygous	This study
41^a^	90/M	Cerebral atrophy	*CNPY3* NM_006586.5	c.275C>T (p. Ser92Leu)	Homozygous/parents heterozygous	This study
42^a^	60/M	Cerebral atrophy	*CNPY3* NM_006586.5	c.329A>G (p.Tyr110Cys)	Homozygous/parents heterozygous	This study
43^a^	90/M	Normal	*SLC12A5* NM_001134771.2	c.518T>C; c.1837C>T (p. Met173Thr; p.Arg613Cys)	Compound Heterozygous/parents heterozygous for each	This study
44^a^	90/M	Normal	*KDM6B* NM_001348716.2	c.909+5G>T (5′ splice site)	Heterozygous/*de novo*	PMID: 2607785
45^a^	90/F	Normal	*MECP2* NM_001110792.2	c.784C>T (p. Arg262Cys)	Heterozygous/*de novo*	This study
46^b^	2/M	Normal	*HUWE1* NM_031407.7	c.9007A>T (p.Ser3003Cys)	Hemizygous, likely *de novo*	This study
47^b^	75/M	Normal	*SCN1A* NM_001165963.4	c.4916G>C (p.Arg1639Pro)	Heterozygous/likely *de novo*	PMID: 29655203
48^b^	1/M	Thin corpus callosum	*GRIN2B* NM_000834.5	c.4102G>T (p.Gly1368Cys)	Heterozygous/likely *de novo*	This study
49^c^	1/M	Cortical tubers	*TSC1* NM_000368.5	c.144+7A>T	Heterozygous and affected father Heterozygous	PMID: 34849272

a In the absence functional evidence and insufficient literature evidence, the variants have been classified as uncertain significance (ACMG criteria: PM2, PP3 for variants *ALG2*, *CNYP3*, *SLC12A5* genes; ACMG criteria PM2, PP3, PS2 for variants in genes *KDM6B*, *MECP2*).

b In the absence of functional evidence and segregation analysis in the parent, the variants have been classified as uncertain significance (ACMG criteria: PM2, PP3).

c Case no. 49, in the absence of functional evidence, the variant has been classified as uncertain significance (ACMG criteria: PM2) and has been previously reported with our collaborators.^13^

**Figure 2:**
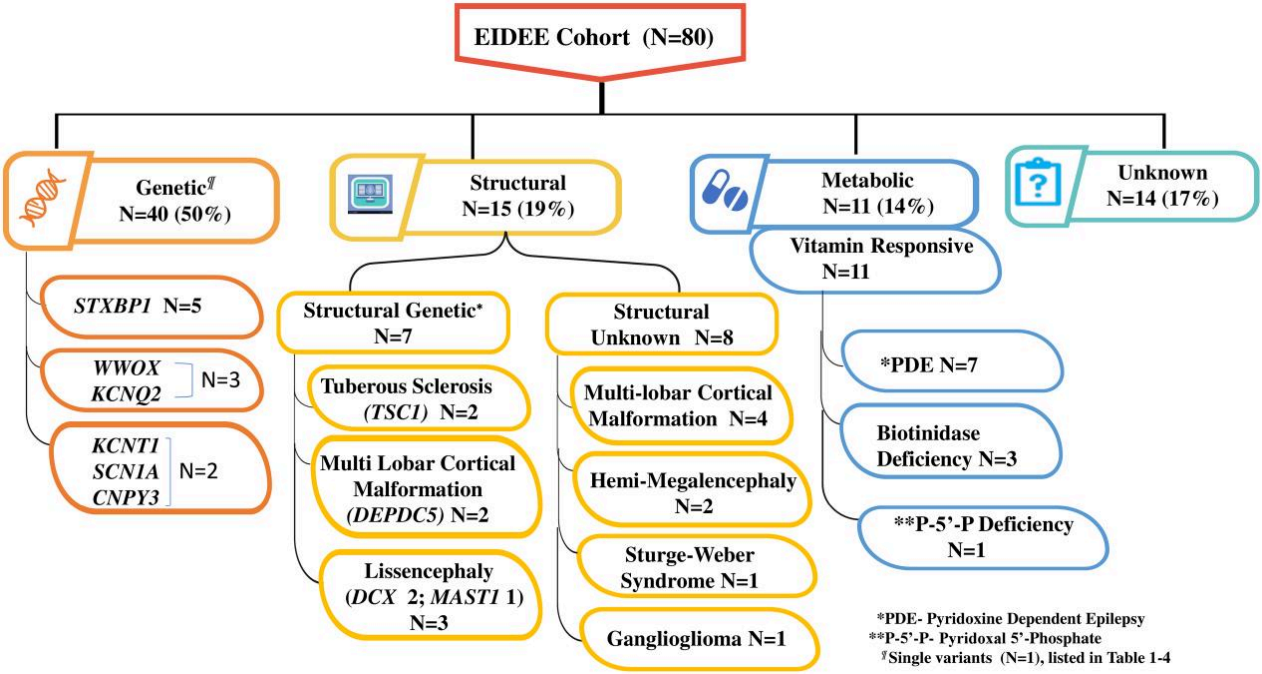
Aetiologies of early-infantile developmental and epileptic encephalopathy (EIDEE), after evaluation. (MRI cartoon in the figure has been downloaded from vectorstock.com@)

The median seizure onset age was 28 days (range, 1–90 days). The male:female ratio was 1.5:1. The median age at the first visit to the paediatric neurologist was 5 months (range, 0.1–162 months), with a median time lag of 3 months (range, 0.1–83 months) from seizure onset to the initial visit. A family history of epilepsy was present in 12 patients (15%). Parents of 10 of these 12 patients were in a consanguineous relationship. After a comprehensive evaluation (Fig. 1), the cause for EIDEE was confirmed in 66 patients (83%), with EIDEE-genetic being the most common aetiological group (Fig. 2). ‘Eleven patients had a metabolic aetiology, and all of these were VR, and therefore, the metabolic group will be considered “EIDEE-VR” from here on in the paper’.

### Baseline clinical features

Seizures were classified mostly based on history and with home videos (39 patients) and/or long-term video EEG monitoring (11 patients), if available.

**Figure 3:**
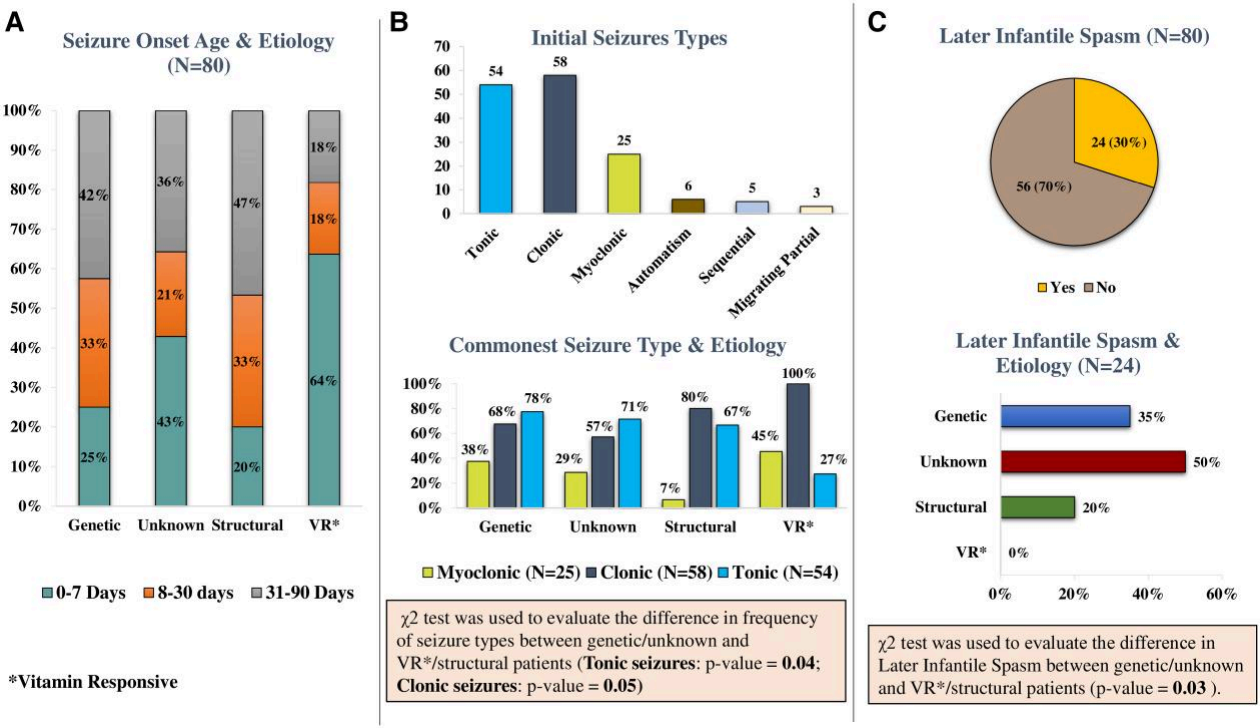
**Seizure characteristics and its relationship with age of onset and aetiological groups.** (**A**) Seizure onset age and aetiology. (**B**) χ^2^ test was used to evaluate the difference in frequency of seizure types between genetic/unknown and vitamin responsive/structural patients (tonic seizures: χ^2^ = 4.26; *P*-value = 0.04; clonic seizures: χ^2^ = 3.80, *P*-value = 0.05). (**C**) χ^2^ test was used to evaluate the difference in likelihood of later infantile spasm, between genetic/unknown and vitamin responsive/structural patients (χ^2^ = 5.02; *P*-value = 0.03).

The most common seizure onset age was after the neonatal period (31/80; 39%), followed by those who had seizures in the initial 7 days (26/80; 32.5%) and the remaining between 8 and 30 days (23/80; 29%). Majority of babies with VR epilepsies (7/11) had their initial seizure in the first 7 days after birth (Fig. 3A). In patients with an underlying ‘genetic’ cause, 75% had seizure onset after first week of birth (Fig. 3A). Clonic seizures, tonic seizures and myoclonic seizures were the most common seizure types (Fig. 3B). Automatism, sequential seizures and migrating partial seizures were uncommon. In 24/80 patients (Fig. 3C), the early seizures had later evolved to infantile spasms (IS) at a median age of 4 months (range, 2–9 months). The first EEG was abnormal in 78/80 patients. ‘The most common EEG abnormalities’ were a burst suppression pattern in 33 patients (42%), multifocal epileptiform discharges in 24 (30%) and hypsarrhythmia in 10 patients (13%).

**Figure 4:**
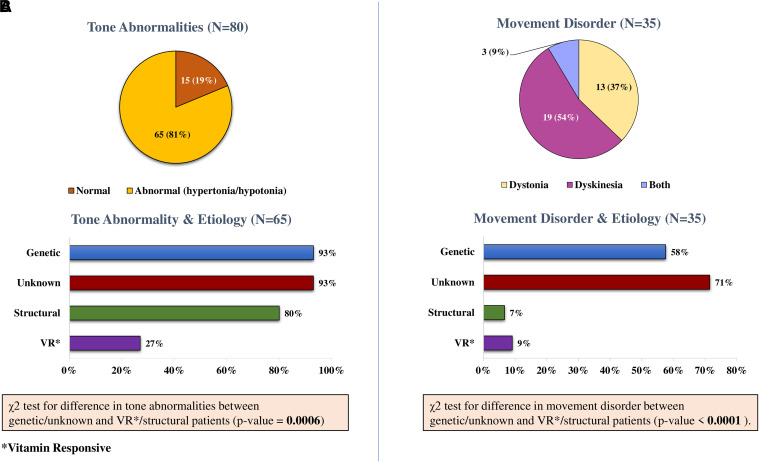
**Comparison of neuromotor issues in aetiological groups.** (**A**) Tone abnormalities: χ^2^ test was used to evaluate the difference in tone abnormalities between genetic/unknown and vitamin responsive/structural patients (χ^2^ = 11.83; *P*-value = 0.0006). (**B**) Movement disorder: χ^2^ test was used to evaluate the difference in movement disorder between genetic/unknown and vitamin responsive/structural patients (χ^2^ = 18.24; *P*-value < 0.0001).

A total of 65 patients (Fig. 4A) had abnormal tone [hypotonic—56 (70%); hypertonic—9 (11%)]. Thirty five patients (44%) also had a movement disorder (Fig. 4B).

### Investigations

#### Initial investigations

‘Metabolic screening’ was done in 41 patients. Three of these 41 patients had abnormal results, subsequently diagnosed with biotinidase deficiency on enzyme assay. ‘Neuroimaging (cranial MRI)’ was abnormal in 35 out of 80 patients (44%) with a malformative lesion seen in 16 (Fig. 2). Remaining 19 patients had non-specific changes on MRI (Tables 1–3 and Table 4).The 16 malformative patients also included a child with *KCNQ2* mutation with a focal cortical dysplasia. However, as he had sequential seizures that were easily controlled with monotherapy with a sodium channel blocker (SCB), he was classified in the EIDEE-genetic group.

**Table 4:** List of copy number variants (CNVs)

S.N	Seizure onset (days)/gender	Neuroimaging	DEE subtype	Technique	Gene	Variant description	Zygosity and segregation	Significance	Literature
50	27/F	Cerebral atrophy	Genetic	cGH array^a^	*WWOX* gene deletion	16q23.1 (78185124–78206556) ×0	Homozygous	Pathogenic	PMID: 33196034
51	45/F	Cerebral atrophy	Genetic	cGH array	Contiguous gene deletion including SCN1A gene (OMIM:619317)	chr2:?_165090150)_166406646_?)del 1	Heterozygous/likely *de novo*^b^	Likely pathogenic	This study
52	45/M	Lissencephaly	Structural-genetic	cGH array	Contiguous gene deletion including DCX gene (OMIM:300067)	Xq23(110084314_110845398) ×0	Heterozygous/likely *de novo*^b^	Likely pathogenic	This study
53	30/F	Normal	Genetic	Cytoarray	Contiguous gene deletion including NHS gene (OMIM: 302350)	Xp22.2p22.13(17194217_18041170) ×1	Heterozygous/likely *de novo*^b^	Likely pathogenic	Overlapping region has been reported

a Comparative genome hybridization.

b Parental testing not done.

#### Genetic evaluation

**Figure 5:**
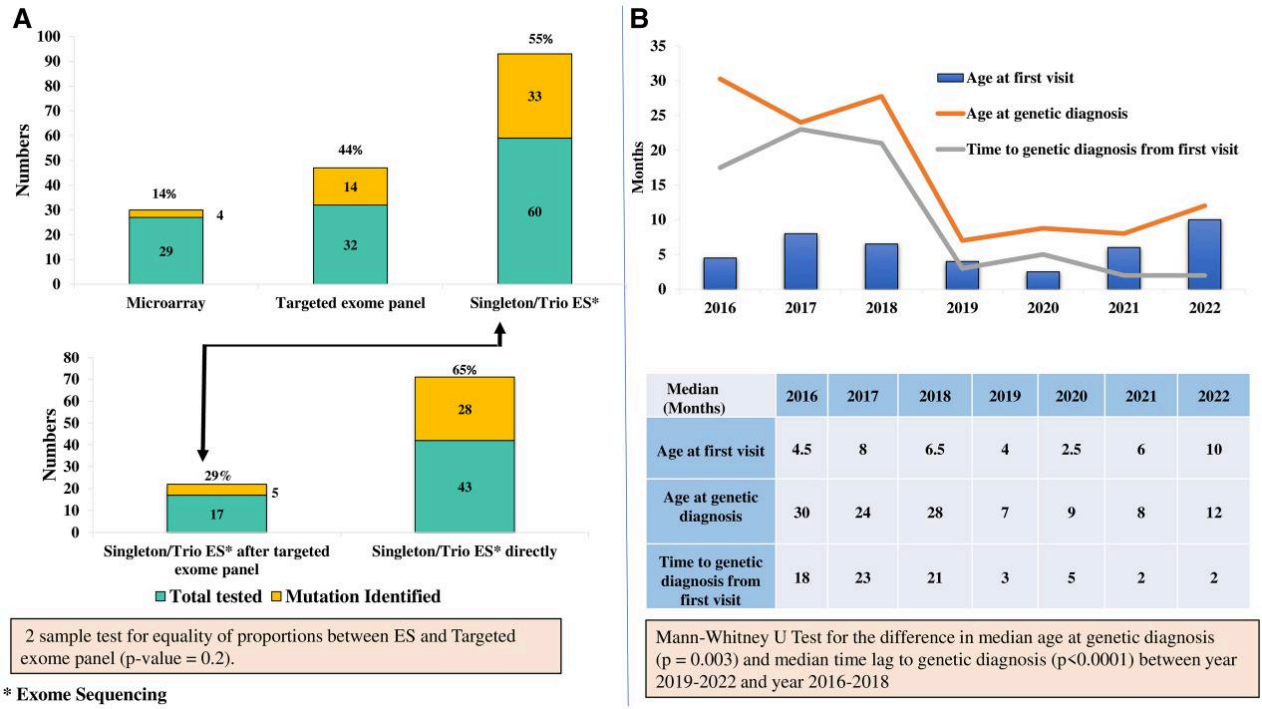
**Comparison of genetic evaluation methodologies and time to reaching a molecular diagnosis over the study period (2016–2022).** (**A**) Yield of genetic evaluation (‘two patients who had Sanger sequencing for tuberous sclerosis genes are not included’). Two-sample proportion test was used to evaluate the difference in effectiveness between singleton/trio exome sequencing and targeted clinical exome panel (χ^2^ = 0.65; *P*-value = 0.2). (**B**) Comparison of time lag to genetic diagnosis and age at genetic diagnosis between year 2019–2022 and year 2016–2018. Mann–Whitney U-test was used to compare the median time lag to genetic diagnosis (*P* < 0.0001) and median age at genetic diagnosis (*P* = 0.003).

Genetic evaluation was performed in 77 patients including 56 trio ES and singleton ES in four (Fig. 5A). The remaining three who could not be tested had either biotinidase deficiency (*n* = 2) or SWS (*n* = 1).

A molecular diagnosis was achieved in 53 out of 77 patients (69%). There were 43 pathogenic or likely pathogenic mutations and 10 VUS matching the clinical profile with 24 novel variants (Tables 1–4). Of the 43 patients with pathogenic/likely pathogenic mutation (Tables 1, 2 and 4), 29 (67%) had autosomal dominant (AD) inheritance and the remaining 14 (33%) had autosomal recessive (AR) inheritance.

In the EIDEE-genetic group, *STXBP-1* was the most common single-gene defect (Fig. 2; Tables 1–3). Of the 11 patients with EIDEE-VR, nine had a genetic evaluation (two families with biotinidase deficiency refused genetic testing). A mutation was identified in six out of these nine patients tested. The three patients with normal genetic evaluation had pyridoxine dependent epilepsy (PDE). In the EIDEE-structural group (*n* = 15), 14 patients had a genetic evaluation (family of one patient with SWS refused testing). Of these 14, a mutation could be identified in seven (47%) patients (Fig. 2).

When the different genetic testing strategies were compared, the difference in yield between singleton ES/trio ES and targeted clinical exome panel (Fig. 5A) was not statistically significant (*P*-value = 0.4). Furthermore, microarray was non-contributory in 86% (25/29) of patients tested (Fig. 5A).

The yield of genetic testing in the neonatal onset EIDEE was 66% (31/47), while in older infants (31–90 days), was 73% (22/30). This difference in yield was not statistically significant (*P*-value = 0.6).

Shorter time lag to genetic diagnosis was seen in patients tested from 2019 onwards as compared to patients tested before 2019 (Fig. 5B), [median difference (months) = 20; *P* < 0.0001]. We also reached a genetic diagnosis at a younger age in children seen from 2019 onwards (Fig. 5B) as compared to patients tested before 2019 [median difference (months) = 15.5; *P* = 0.001].

### Follow-up outcome

**Figure 6:**
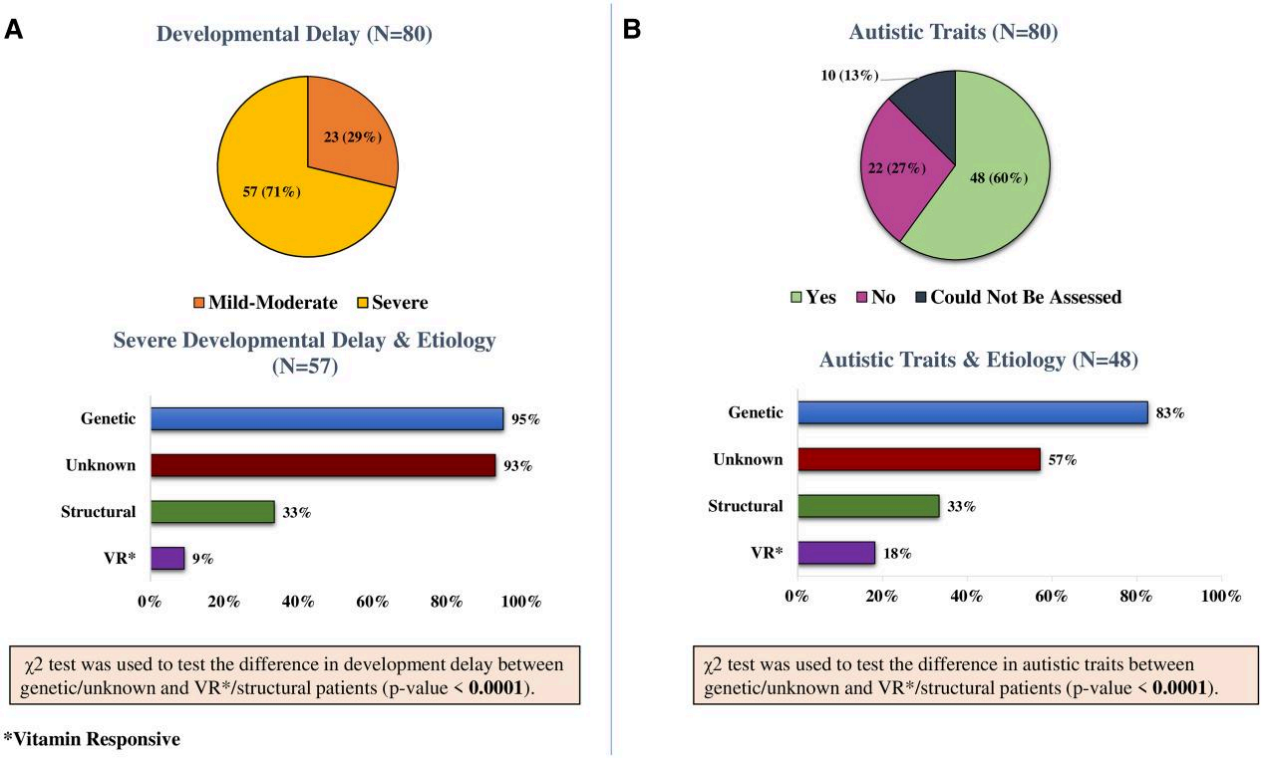
**Comparison of neurodevelopmental profile of the aetiological groups.** (**A**) Developmental delay/intellectual disability: χ^2^ test for difference in development delay/intellectual disability between genetic/unknown and vitamin responsive/structural patients (χ^2^ = 40.22; *P*-value < 0.0001). (**B**) Autistic behaviour: χ^2^ test for difference in autistic behaviour between genetic/unknown and vitamin responsive/structural patients (χ^2^ = 15.58; *P*-value < 0.0001).

The mean follow-up period was 30 months (range, 4–72 months). Most children (71%) had severe DD/ID (Fig. 6A). Majority of patients also demonstrated autistic behaviour (Fig. 6B). In follow-up, 11 children (14%) had died (five due to drug-resistant repetitive seizures and two following respiratory illness; in four patients the reason for death could not be confirmed).

**Figure 7:**
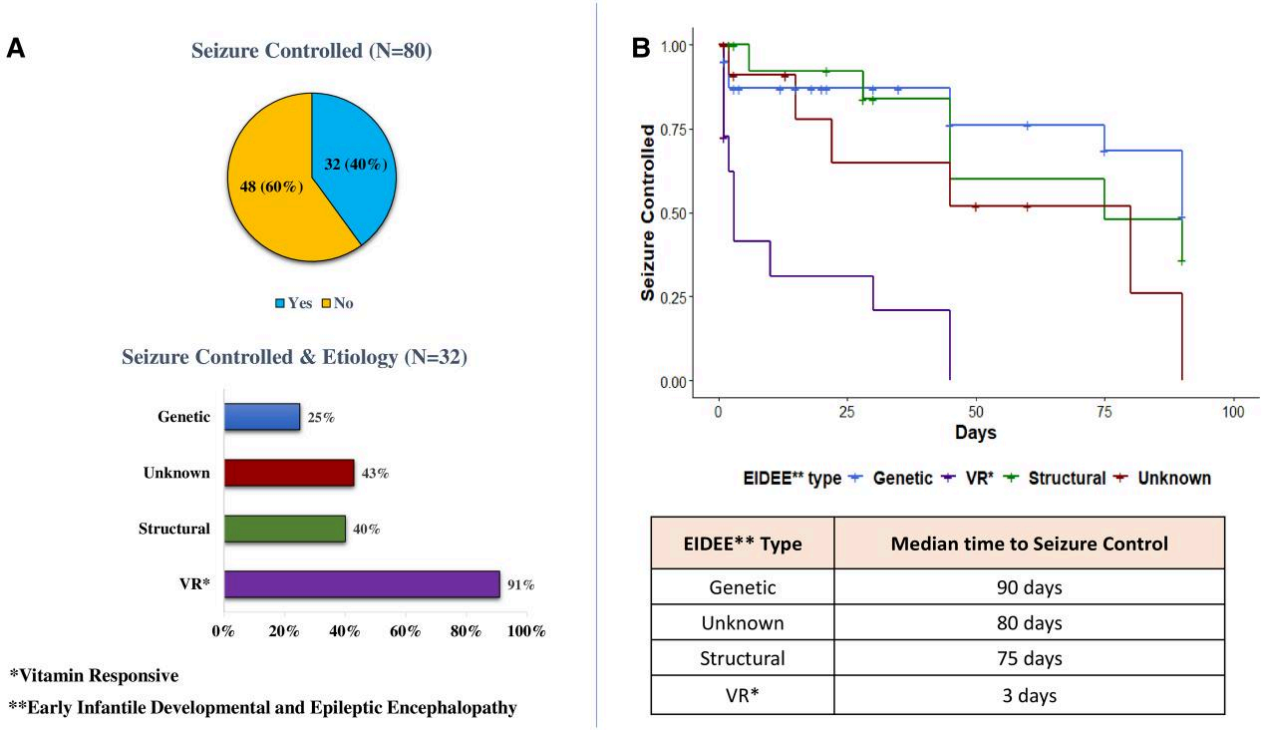
**Epilepsy outcome.** (**A**) Seizure outcome at follow-up in whole cohort and the aetiological groups: χ^2^ test for difference in seizure control between genetic/unknown and vitamin responsive/structural patients (χ^2^ = 6.18; *P*-value = 0.01). (**B**) Kaplan–Meier curve (KM) to compare probability of seizure control for the aetiological groups. In seizure-free patients, the table below KM curve presents the median time to seizure control in the different aetiological groups.

At last follow-up contact, 60% patients still had drug-resistant epilepsy (Fig. 7A). A median of five ASMs (range, 1–10) was used for seizure control. In patients who were seizure-free, control was achieved at a median of 10 months of age (range, 0.1–72 months). The seizure cessation was more likely in the EIDEE-VR (9/11 patients—6/7 PDE, 3/3 biotinidase deficiency). In eight of these nine seizure-free patients, ASMs had also been weaned off.

Of the 15 patients with EIDEE-structural aetiology, 11 had drug-resistant epilepsy. After presurgical evaluation, epilepsy surgery was offered to seven patients of whom six families agreed (three each had hemispherectomy and multilobar resection). The median age of epilepsy surgery was 18 months (range, 9–36 months). Three of these six operated patients were seizure-free at the last follow-up visit. Two patients with germline mutation (one each with *TSC1* and *DEPDEC5*) who were operated due to drug-resistant focal epilepsy continued to have frequent seizures, post-surgery.

Multivariate logistic regression was applied to investigate the impact of independent variables, namely, age of seizure onset, time lag of the first visit to a paediatric neurologist and the underlying EIDEE aetiology, on the dependent variable, i.e. seizure control. Among all these factors, only a diagnosis of EIDEE-VR had a statistically significant positive effect on seizure control (*P* = 0.02).

#### Comparison of aetiological groups

##### Comparison of seizure characteristics and seizure outcomes

When aetiological groups were compared for the frequency of the three most common seizure types (clonic, tonic and myoclonic seizures), clonic seizures were more frequent in VR/structural aetiologies [Fig. 3B; Risk Ratio (RR) = 1.36; *P* = 0.05] as compared to genetic/unknown groups. On the contrary, VR/structural patients were less likely to have tonic seizures (RR = 0.66; *P* = 0.04). In children in the genetic/unknown groups, the initial seizure types were more likely to later evolve to IS (Fig. 3C; *P* = 0.03). Furthermore, the comparison of probability of seizure-free duration for the four aetiological groups was presented using the Kaplan–Meier curve (Fig. 7B). Among the patients who had achieved seizure freedom, the median time to seizure control was 3, 75, 80 and 90 days, respectively, for VR, structural, unknown and genetic causes.

##### Comparison of neuromotor deficits and neurodevelopmental outcome

Babies with genetic/unknown aetiology were more likely to have tone abnormalities [Fig. 4A; 50 (93%)] as compared to VR/structural patients [15 (58%)], a statistically significant difference [Odds Ratio (OR) = 9; *P* = 0.0006]; the movement disorder (Fig. 4B) was also more common in the genetic/unknown groups (OR = 19; *P* < 0.0001).

Children in the genetic/unknown groups had a higher probability of having severe DD/ID (Fig. 6A) as compared to the VR/structural patients (OR = 57; *P* < 0.0001). Similarly, patients in the genetic/unknown groups were more likely to have autistic traits (Fig. 6B, OR = 37; *P* < 0.0001).

### Impact of genetically informed management

Achieving a genetic diagnosis was helpful in better treatment guidance in seven (9%) of the 77 patients tested. Sodium channel blockers (SCBs) were effective in six (*SCN1A 1*, *KCNQ2 2*, *FGF12 1*, *SCN8A 1* and *SCN2A 1*) and quinidine in one of the two patients with *KCNT1* mutation (the second family could not procure quinidine from overseas, as this medication is not available in India). On the contrary, in three patients with PDE, a normal genetic evaluation prompted us to discontinue pyridoxine, resulting in seizure recurrence in all three. One of the patients with *KCNQ2* mutation, continued to have drug-resistant seizures even after an adequate trial of multiple SCBs.

## Discussion

The DEE, particularly EIDEE, is a heterogeneous group of severe epilepsies with a broad range of underlying aetiologies including structural brain malformations, vitamin responsive epilepsies and single-gene defects.^14^ In the present study, not only the ‘genetic causes’ but also the whole aetiological landscape of EIDEE including the structural and metabolic aetiologies have been evaluated and were compared for phenotypic differences. For this, we prospectively collected the clinical data to understand the phenotypic spectrum in each aetiological group and subsequently performed a comprehensive metabolic, neuroradiological and genetic evaluation. As this study was done at a single centre, the patient profile, clinical assessment, testing protocol and follow-up evaluation were all homogenous. The follow-up data were available for up to 6.5 years from the initial visit, which was helpful in confirming the long-term neurodevelopmental and epilepsy outcome.

After comprehensive evaluation, an aetiology could be established in majority of our patients. Of these, single-gene disorders were the most common underlying cause. Similar studies, on the whole spectrum of aetiologies of EIDEE, have not been published for a useful comparison. A Scottish epidemiological study on causes of early childhood epilepsies has identified an aetiology in 76% of its 17 EIDEE patients, with monogenic disorders being the commonest group.^15^

One of the aims of this study was also to assess if there were clinical clues for differentiating the aetiologies of EIDEE. We observed some important distinctions between patients in genetic/unknown groups as compared to patients in the remaining aetiologies (comprising VR/structural causes in our cohort).

Children in the genetic/unknown groups were more likely to have severe DD/ID and autistic traits. Similarly, on motor assessment, the genetic/unknown groups more often had tone abnormalities, particularly central hypotonia and a dyskinetic movement disorder. This phenotypic presentation of severe DD/ID, central hypotonia and movement disorder has also been previously reported extensively in the EIDEE-genetic patients.^14–17^ On the contrary, patients with EIDEE-VR had shown minimal to no neurological dysfunction, particularly if specific treatment was started early, an observation that has also been confirmed previously.^18,19^ Similarly, focal nature of the lesion, including some who also benefited from early epilepsy surgery, could have accounted for lesser morbidity in EIDEE-structural patients as compared to genetic/unknown aetiologies.

The early (‘within 7 days of birth’) onset of seizures in most EIDEE-VR patients was also a helpful diagnostic clue. The age of onset of seizures was though not helpful in clinically differentiating the remaining causes.

There was also a clear distinction between initial seizure type and underlying aetiology. Tonic seizures were more common in genetic/unknown patients, while clonic seizures were more frequent in patients in the VR/structural groups. Though only limited published data are available on seizure semiology differentiating the different aetiological groups, both clonic and tonic seizures have been frequently reported in EIDEE patients.^20,21^ Another interesting observation was the frequent evolution of these early seizures to IS in patients with a genetic/unknown aetiology. The frequent occurrence of IS is not unusual and has been commonly reported in patients with ‘genetic’ DEE.^12,22–24^ However, IS was infrequent in patients with structural/VR epilepsies. In EIDEE-structural group, the median age of surgery was after the age of one year. Hence, in these patients, it is more likely that the focal lesion and not the surgical intervention would have been the reason for infrequent progression to IS. In VR epilepsies, the early identification of the cause could have helped to prevent the progression of initial seizures to later IS. But in this study as early seizures in only a small number of patients from the whole cohort had evolved to IS, it is difficult to make a definitive conclusion about this observation.

Therefore, the above association of seizure types and aetiologies could help in predicting the underlying cause: predominant clonic seizures favouring VR (‘particularly when onset was within the first week after birth’) or the structural cause, and tonic seizures that later evolved to IS suggesting a ‘genetic/unknown’ aetiology.

We had initially investigated most of the patients with neuroradiological, neurophysiological and if required metabolic testing. Subsequently, a detailed stepwise genetic testing was done with NGS and if indicated, microarray. Consequently, another important aim of our study was to confirm the diagnostic yield and utility of these investigations.

It was observed that nearly all patients had ‘EEG’ abnormalities; EEG findings though were not helpful in pointing to a specific aetiology. ‘Neuroimaging’ was also more often normal or non-diagnostic in our patients. But still, it was a useful first-line investigation for classifying the patients into aetiological groups and for treatment planning. The presence of a malformative/dysplastic lesion was helpful in guiding further evaluation beyond ASMs. In fact, epilepsy surgery was helpful in controlling seizures in some of our patients. Likewise, ‘metabolic investigations’ were also mostly non-diagnostic, the exception being three patients with biotinidase deficiency. In these three patients also, the metabolic results had only reconfirmed the strong clinical suspicion of biotinidase deficiency.

Among all the investigations, ‘the genetic evaluation’ had the best yield and was diagnostic in 69% of our EIDEE patients. The higher yield of genetic testing is not surprising in view of early onset of these severe epilepsies. In published literature, genetic evaluation of DEE has had a variable yield of 27–53%.^4–9,25–27^ These studies incorporated varying testing strategies from targeted gene panels and ES with copy number variations (CNVs) to whole genome sequencing and array cGH. A study published from the Indian subcontinent in which DEE patients had a comprehensive genetic evaluation also had a lower diagnostic yield as compared to this study.^24^ The prospective nature of our study that recruited only patients with early onset severe epilepsy, where a ‘genetic’ cause is more likely, helped us to achieve a higher yield as compared to some of the previous studies. In our cohort, there were excess of *de novo* (AD) variants. These results are comparable with other studies on paediatric refractory epilepsies/epileptic encephalopathies in which majority of patients with significant variants also had *de novo* mutations.^28,29^

The yield of genetic evaluation was similar in neonatal and post-neonatal onset EIDEE. This was contrary to the previously published studies in which genetic evaluation was more often diagnostic in patients who have had seizure onset in the neonatal period.^6,9^

The diagnostic yield of singleton ES/trio ES and targeted clinical exome panel of proband (followed by parental testing) was comparable in our cohort. In patients with non-diagnostic NGS, we could identify further three more patients on microarray. A study on NGS-negative patients had reported a low yield for pathogenic CNVs in DEE.^26^ Even though the yield of microarray was limited in EIDEE, until genome sequencing replaces ES and microarray as the standard first-line genetic test, in ES negative patients, microarray could still be an important next step to further improve the diagnostic yield.

The underlying genetic causes are heterogenous in this study. In the EIDEE-genetic group, *STXBP-1* was the most common gene mutation, a commonly reported monogenic cause of EIDEE.^6,23,27^ WWOX has been increasingly recognized as an important cause of EIDEE,^30,31^ as was also seen in some of our patients.

In addition to the significant variants (pathogenic and likely pathogenic variants; Tables 1, 2 and 4), rare, conserved amino acids in disease genes were detected in 10 more cases (Table 3). Though these variants may be probably significant, however due to insufficient literature evidence or unavailability of functional evidence or the absence of segregation analysis in the family, and/or partial clinical match, their effect on the disease phenotype remains unknown. These were classified as VUS according to the ACMG guidelines.^11^ Three (3/10) had rare homozygous missense variants; two with variants in *CNPY3* gene associated with DEE; a case with *ALG2* gene associated with congenital disorder of glycosylation. Another case with compound heterozygous variant in *SLC12A5* gene that is commonly associated with DEE was also detected. These variants had a minor allele frequency of <0.001% or absent in the population databases and predicted to be damaging by two or more ‘*in silico*’ tools. In two cases, *de novo* variants in genes *MECP2* and *KDM6B* that match partially with the patients’ clinical phenotype were detected. In the absence of functional evidence, they also remain classified as uncertain significance. In the absence of segregation analysis in the parents, the clinical significance of the conserved missense variants in *SCN1A* (p.Arg1639Pro) and *GRIN2B* (p.Gly1368Cys) detected remains unknown. Therefore, these two were also classified as VUS. Reclassification of all the above variants could be possible in future, based on literature evidence or variant functional analysis that may become available.

In the EIDEE-structural group, we were able to detect causative variants in *DCX (2)*, *DEPDC5* (2), *TSC1(2)* and *MAST1* (1) genes, explaining the malformative MRI findings in nearly half of the patients. One of our patients with *KCNQ2* mutation had left frontal dysplasia, hitherto unreported. This child had early neonatal onset epilepsy with sequential seizures and a generalized bust suppression pattern on EEG. The seizures were also well controlled on a single SCB, more in keeping with a genetic (channelopathy) rather than a structural aetiology.

The metabolic aetiologies were not common in our patients. We had excluded IEM patients who had presented with acute reactive seizures, which could have contributed to rarity of these disorders in our cohort. Non-ketotic hyperglycinaemia, molybdenum cofactor/isolated sulphite oxidase deficiency, glucose transporter defect-1 (GLUT-1) and serine biosynthesis defects have often been reported as metabolic causes of EIDEE.^32^ None of our metabolic patients though were diagnosed with any of the above disorders. Like our observation, these disorders though often mentioned have been a rare cause of EIDEE, in populations with both similar and different demographics as our cohort.^15,24^ In fact, all our EIDEE-metabolic patients had VR epilepsy, a potentially treatable cause of EIDEE with good long-term neurodevelopmental and epilepsy outcome. Our data reiterate that if a child presents with severe epilepsy in first three months of life, more so in the first week of life with no identified cause from history or early investigations (specifically metabolic screen and neuroimaging), a trial of pyridoxine is warranted.

Another interesting finding in our study was the time lag to identifying a causative mutation when comparing patients tested before and since 2019. When we followed the stepwise approach (‘before 2019’) of doing targeted clinical exome panel before singleton ES/Trio ES, as compared to singleton ES/Trio ES directly (‘from 2019 onwards’), there was a significant delay in reaching a molecular diagnosis. Another factor that could have contributed to the earlier diagnoses since 2019 could be the better awareness and easier accessibility of NGS, prompting the clinician to request genetic testing earlier during the evaluation of patients with EIDEE. A study was done recently to determine the cost-effectiveness of early genetic testing with targeted-whole exome sequencing (WES).^33^ They concluded that the use of targeted-WES yields diagnoses earlier and at a lower cost, an observation that we could also confirm in our patients.

Even though the rapidly advancing field of genomics-based therapy can alter the course of these severe epilepsies in future, the molecular diagnosis was found to be helpful only in guiding treatment in some of our patients. On the contrary, in our patients with PDE for whom pyridoxine was discontinued (following a normal genetic evaluation), there was seizure recurrence, requiring reinitiation of pyridoxine. There is a possibility that deep intronic mutations in the *ALDH7A1* gene might have been missed on exome sequencing, or other unknown genes are also involved in PDE. In addition, in two of the patients with *DEPDEC5* mutation, despite the focal nature of epilepsy and associated brain malformation, we were hesitant to suggest epilepsy surgery. After failed attempts with multiple ASMs, resective surgery was performed on one of them. The delay could have resulted in failure of surgery and associated significant neurodevelopmental delay, which could have been possibly prevented if we had intervened earlier. Nevertheless, confirmation of the underlying genetic cause does help the families in ending the long ‘diagnostic journey’. This was also confirmed in a recent study where reaching a genetic diagnosis helped families to cope and adapt better, thus improving their quality of life.^34^

There were some limitations to our study. This is not a population-based study, hence, bias is inevitable, as reflected in greater number of males seen. This was a possible selection bias, as in our culture, the male child is given preferential treatment and more likely to be taken to a tertiary hospital. There could also have been a potential referral bias as patients with more severe epilepsy were being referred to us as costs of treatment were borne by families, at our hospital. The phenotypic evaluation also had some inherent biases. Our assessment of seizure characteristics was mostly based on clinical history because only few home videos and long-term video EEG recordings were available. Therefore, automatisms, sequential seizures and migrating partial seizures could have been under-represented in this cohort. A significant number of patients also could not co-operate for formal psychometric testing as they had profound neurodisabilities. In these patients, the assessment was mainly based on the evaluation of the treating paediatric neurologist. In addition, it was difficult to differentiate behaviours of a child with severe to profound delay from autistic behaviours and therefore, autism may have been over-diagnosed.

Despite these limitations, there were some important and useful observations made in our study. Following a comprehensive evaluation, we were able to confirm the underlying aetiologies in most of our EIDEE patients. Our study also provides some insights into the differentiating features of the common aetiological groups of EIDEE. Patients with genetic/unknown aetiologies particularly were more likely to have a significant long-term morbidity in form of severe DD/ID, autistic behaviour, tone abnormalities and movement disorder. Among these patients, the ‘genetic’ EIDEE patients were also more likely to have drug-resistant epilepsy. On the contrary, EIDEE-VR patients, if diagnosed and treated early, had a good outcome.

## Supplementary Material

fcad243_Supplementary_Data

## Data Availability

The data for this study are available from corresponding author on reasonable request.
