# Supplementary material for: Early-infantile developmental and epileptic encephalopathy: the aetiologies, phenotypic differences and outcomes—a prospective observational study
Source: Brain Commun. 2023 Sep 10;5(5):fcad243. doi: 10.1093/braincomms/fcad243 (PMC10702464; doi:10.1093/braincomms/fcad243)
Supplement: fcad243_Supplementary_Data [file fcad243_Supplementary_Data.docx]

**TEST METHODOLOGY**

**NEXT GENERATION SEQUENCING (NGS):**

**DNA isolation, Exome library preparation and sequencing:**

Genomic DNA isolated from whole blood using QIAamp DNA Blood Mini Kit (Qiagen, Germany) is quantified using Qubit fluorometer (Thermo Fisher Scientific, USA). For library preparation, 200ng of the Qubit quantified DNA is fragmented to ~350bp inserts. The library is hybridised and enriched using whole exome probes. The fragments are end-repaired, 3′ adenylated and ligated with the indexed adapters. The adapter-ligated fragments are then amplified with adapter-specific primers followed by size selection and purification to generate gDNA library. The generated library is assessed for fragment size distribution using Tape Station (Agilent, USA) and quantified using Qubit (Thermo Fisher Scientifc, USA). The library is sequenced as 2 × 150 bp paired‐end reads on an Illumina HiseqX / Novaseq (Illumina, CA) machine according to the manufacturer's protocol to an average sequencing depth of ≥80-100x.

**Data processing, variant calling and annotation:**

Following quality check and adapter trimming using fastq-mcf (version 1.04.676), the sequencing reads obtained are aligned to human reference genome (GRCh38.p13) using BWA.^1^ The aligned reads are sorted, and duplicate reads removed. Single nucleotide variants (SNVs) and small Indels variants are called using GATK best practices pipeline using Sentieon (v201808.07).^2^ Gene annotation of the variants is performed using VEP program against the Ensembl release 99 human gene model. ^3^ The variants are annotated for allele frequency [population databases GnomAD (v2.10), GnomAD (v3.0), 1000 genome, MedGenome population specific database], *in silico* prediction tools [PolyPhen‐2, SIFT, Mutation Taster2, and LRT] and disease databases [OMIM, ClinVar and HGMD]. The clinically significant variants are sequentially prioritised based on a) minor allele frequency; b) previously reported with disease literature; c) supporting damaging effect by ≥ 2 *in silico* prediction tools; d) clinical features of the proband and analysed using Varminer (MedGenome proprietary variant interpretation tool)^1^. In brief the data was prioritised for clinically relevant genes a) genes previously associated with disease phenotype (OMIM, Clinvar); b) Variants with population frequency less than 5% databases (gnomAD, 1000 genome, Internal population specific database); c) Variants predicted to alter protein sequence (missense, nonsense, frameshift, splice site, and short indel variants; canonical splice variants and copy number variants. The variants were then interpreted based on the American College of Medical Genetics 2015 guideline^4^ as pathogenic (previously in a similar phenotype, loss of function variants in genes with known mechanism) or likely pathogenic (the variant may not be previously reported but the gene is well associated with the phenotype, conserved and damaging effect supported by prediction tools) matching the patient’s phenotype and disease mode of inheritance.

In addition, variants of unknown significance (VUS) in both autosomal dominant and recessive genes completely or partially matching the clinical profile of the patient were considered to understand the underlying genetic etiology.

Copy number variants (CNVs) were detected from targeted sequence data using the ExomeDepth (v1.1.10) method. Based on the comparison of read-depths of the test data with the matched aggregate reference dataset, the algorithm detects CNVs (≥ 400bp deletions and duplications).^5^

**Microarray:**

Twenty nine patients who remained without a genetic diagnosis after NGS, were processed for *array* [*comparative genomic hybridization*](https://www.sciencedirect.com/topics/medicine-and-dentistry/comparative-genomic-hybridization) *(aCGH) analysis* (Affymetrix CytoScan™ 750 K; N=9) or *Global Screening Array with Cytogenetics* (GSACyto; N=20) and analysed on Bionano’s NxClinical Analysis Software. Copy number variants overlapping with significant disease causing variants and rare CNVs not overlapping with healthy individual data base were prioritised and analysed based on the clinical phenotype. The relevance of the variants were assessed based on databases: DECIPHER (<https://decipher.sanger>. ac.uk/), ISCA consortium (<https://www.iscaconsortium.org/>), OMIM (Online Mendelian Inheritance in Man,

https://www.omim.org/),UCSC genome browser (http:// genome.ucsc.edu/), and PubMed (<http://www.ncbi.nlm.nih>. gov/pubmed)

The significant variants in genes correlating the disease symptoms/phenotype and inheritance (*de novo*, autosomal dominant and autosomal recessive) are prioritised. The significance of the prioritised variants (SNVs and CNVs) are assigned based on ACMG guidelines.^4^

**Notes:** The *in-silico* predictions are based on Variant Effect Predictor (v104), [SIFT version - 5.2.2; PolyPhen - 2.2.2; LRT version (November, 2009); CADD (v1.6); Splice AI; dbNSFPv4.2] and MutationTaster2 predictions are based on NCBI/Ensembl 66 build (GRCh38 genomic coordinates are converted to hg19 using UCSC Lift Over and mapped to MT2). Diseases databases used for annotation includes ClinVar (updated on 5082021), OMIM (updated on 5082021), HGMD (v2021.3), LOVD (Nov-18), DECIPHER (population CNV) and SwissVar.

**Supplementary references**

1. Li H, Durbin R. Fast and accurate long-read alignment with Burrows-Wheeler transform. Bioinformatics. 2010; 26:589-95.
2. Freed D, Aldana R, Weber JA, Edwards JS. The Sentieon Genomics Tools–A fast and accurate solution to variant calling from next-generation sequence data. BioRxiv. 2017 Mar 10:115717.
3. McLaren W, Pritchard B, Rios D, Chen Y, Flicek P, Cunningham F. Deriving the consequences of genomic variants with the Ensembl API and SNP Effect Predictor. Bioinformatics. 2010; 26:2069-70.
4. Richards S, Aziz N, Bale S, et al. ACMG Laboratory Quality Assurance Committee. Standards and guidelines for the interpretation of sequence variants: a joint consensus recommendation of the American College of Medical Genetics and Genomics and the Association for Molecular Pathology. Genet Med. 2015; 17:405-24.
5. Plagnol V, Curtis J, Epstein M, et al. A robust model for read count data in exome sequencing experiments and implications for copy number variant calling. Bioinformatics. 2012; 28:2747-54.
